# On the Performance of Trimetazidine and Vitamin E as Pharmacoprotection Agents in Cyclosporin A-Induced Toxicity

**DOI:** 10.1155/2013/605640

**Published:** 2013-04-11

**Authors:** De la Cruz Rodríguez Lilia Cristina, Rey María del Rosario, Araujo Carmen Rosa, Oldano Ana Veronica

**Affiliations:** ^1^Clinical Biochemistry III, Instituto de Bioquímica Clínica, Facultad de Bioquímica Química y Farmacia, Universidad Nacional de Tucumán, Balcarce 747, Tucumán, 4000 San Miguel de Tucumán, Argentina; ^2^Instituto de Bioquímica Clínica, Facultad de Bioquímica Química y Farmacia, Universidad Nacional de Tucumán, Balcarce 747, Tucumán, 4000 San Miguel de Tucumán, Argentina

## Abstract

The immunosuppressant drug cyclosporin A (CyA) has been used in diseases with immunological basis and in transplant patients. Nephrotoxicity and hepatotoxicity are the main adverse effects of this drug. To find a protective drug against those effects we assayed the cardioprotector Trimetazidine (TMZ) and vitamin E, used as nutritional supplements to alleviate oxidative stress. Six groups of eight male Wistar rats each were prepared (groups A–F): A, control; B, vitamin E (10 mg/Kg/day); C, TMZ (20 mg/Kg/day); D, 25 mg/Kg/day CyA; E, CyA and vitamin E (25 mg/Kg/day CyA + 10 mg/Kg/day Vit E); F, TMZ for 20 days (20 mg/kg/day); and then CyA (25 mg/kg/day) and TMZ (20 mg/Kg/day). The experiment lasted 120 days. The exposure of rats to CyA promoted nephrotoxicity and hepatotoxicity with an increase in serum urea, creatinine, and glutamate dehydrogenase (GLDH). Structural and ultrastructural studies of liver and kidney were performed. Group D showed adverse effects induced by CyA since statistically significant differences were found with respect to the control group (A). Vitamin E (E) showed no protective effect. Pretreatment with TMZ (F) attenuated the adverse effects of CyA. We conclude that CyA-induced nephrotoxicity and hepatotoxicity are attenuated by the cytoprotective effect of TMZ. TMZ inhibits the reabsorption and, consequently, the accumulation of CyA in the cell. The antioxidant capacity of vitamin E did not improve the effect of CyA.

## 1. Introduction

Cyclosporin A (CyA) is a cyclic undecapeptide with strong immunosuppressive activity, since it has a specific inhibitory effect on the T-cell receptor signal transduction pathway [1, 2]. Reactive oxygen species (ROS) have been implicated in the nephrotoxicity and hepatotoxicity caused by CyA, either by direct action or by the activation of lipid peroxidation (LP) [3–6]. 

Our previous work showed that the effect of CyA on the antioxidant defense system (ADS) is related to lipoperoxidation and liver function. The results for the chronic and acute treatment, only with 20 mg/Kg/day of CyA, caused alterations in liver parenchyma histoarchitecture [7]. In previous works we showed that CyA administration to organ transplant patients caused an increase in the levels of serum creatinine and urinary excretion of gamma-glutamyl transpeptidase (uGGT). These functional changes were dose and time dependent, showing structural alterations with doses higher than 15 mg/Kg/day CyA in chronic treatments [8]. Morphofunctional alterations in the liver parenchyma were observed in rats treated with CyA at different doses for 120 days. Doses higher than 15 mg/Kg/day CyA, equivalent to concentrations in blood of 437 ± 13.40 ng/mL or greater, caused functional alterations in complexes I and II of the mitochondrial respiratory chain in the liver of treated rats [9].

In an attempt to design and develop therapeutic strategies that could prove effective against the adverse effects induced by CyA, some investigators explored the protective role of antioxidants [10]. Vitamin E (Vit E) is the collective name for a group of liposoluble compounds derived from tocol and tocotrienol, alpha tocopherol being the one with greatest biological activity. Its absorption and distribution are closely related to the digestion, absorption, and distribution of lipids, the liver being its main storage organ, from where it is rapidly mobilized. The characteristic of “biological antioxidant” of Vit E derives from its molecular structure as its capability of fixing ROS types O_2_^−^, O_2_^2−^, and OH^−^ [11, 12]. Vitamin E prevents oxidation of polyunsaturated fatty acids and lipoproteins and it has been shown that the antioxidant effect of Vit E might be beneficial in the prevention of autoimmune, inflammatory and infectious diseases as well as neoplasia [13–15].

Trimetazidine (TMZ), 1-2,3,4 trimetoxibencil piperazine, is a drug used as a cardioprotector since it prevents the secondary cell death that usually follows transient myocardial ischemia [16]. TMZ is a cytoprotector whose place of action and the mechanism and chronological order of its effects are not yet known in depth. According to some authors, TMZ acts at the mitochondrial level diminishing the beta-oxidation of fatty acids. Another beneficial effect described in the existing literature is the attenuation of intracellular changes of sodium and hydrogen ions. Numerous studies have analyzed the protective effect of TMZ against myocardial ischemia-reperfusion injury in different models. Among the mechanisms suggested, the following have been mentioned as the most probable: hemodynamic changes, reduction in ROS toxicity, decrease in the inflammatory reaction, optimization of the energetic metabolism, and reduction in the utilization of fatty acids in favor of carbohydrates [17–20].

 Our experiments using an *in vivo* model to study the effect of TMZ on the nephrotoxicity induced by gentamicin demonstrated that TMZ, when used in a 7-day pretreatment, was able to attenuate the nephrotoxic effect of this aminoglycoside [21].

To find a protective drug against the toxicity induced by CyA during chronic treatment, we assayed the cardioprotector Trimetazidine (TMZ) and vitamin E, used as nutritional supplements to alleviate oxidative stress.

## 2. Materials and Methods

### 2.1. Animals and Drugs

The experiments were performed with adult male Wistar rats of 180 to 200 g body weight housed in standard cages with a 12 h light-dark cycle (light on at 7:00 a.m.) at 20°C and 60% humidity. The animals were given a standard diet for rodents and tap water. 

All experimental procedures complied with the regulations of the European Union (86/60/EEC) and with the recommendations of the Federación de Sociedades Sudamericanas de la Ciencia de Animales de Laboratorio-FESSCAL (Federation of South American Societies of Laboratory Animal Science).

- In the treatment of the animals the following drugs were used: CyA provided by Sandoz Laboratories (Germany) Sandimmune.
- Vitamin E provided by Raymos Laboratories, Tanvimil E 400 mg.
- Trimetazidine provided by Servier Laboratories, Vastarel 20 mg.

### 2.2. Experimental Design

The animals were randomly divided into six groups (*n* = 8). The groups were treated according to the following schedule.

-  Group A (control): animals were fed a standard rat-mouse chow for 120 days.
-  Group B (control Vit E): animals were fed a standard rat-mouse chow supplemented with 10 mg/Kg/day Vit E for 120 days.
-  Group C (TMZ control): animals were fed a standard rat-mouse chow supplemented with 20 mg/Kg/day TMZ for 120 days.
-  Group D (CyA control): animals were fed a standard rat-mouse chow supplemented with 25 mg/Kg/day CyA, the drug being orally administered for 120 days.
-  Group E (CyA + Vit  E): animals were fed a standard rat-mouse chow treated with 25 mg/Kg/day CyA and 10 mg/Kg/day Vit E, the drugs being orally administered for 120 days.
-  Group F (CyA + TMZ): animals were fed a standard rat-mouse chow pretreated with 20 mg/Kg/day TMZ for 20 days and then treated with 20 mg/Kg/day TMZ and 25 mg/Kg/day CyA for 120 subsequent days.

During the chronic experiment, the animals were studied with daily evaluations of behavior, appetite, and activity.

The body weight of the animals was determined at three points in time: the beginning of the experiment, 60 days after the beginning, and 120 days after the beginning. At the end of the experiment, the liver and kidney were excised and weighed.

### 2.3. Samples

At the beginning and at the end of the experiment, blood samples were collected by tail vein punction and by intracardiac punction, respectively, for the biochemical studies.

### 2.4. Methods

The nitrogen compounds in blood, urea, and creatinine were determined using the urease method and Jaffe's colorimetric method, respectively. The reactives were provided by Wiener Laboratories [22, 23].

In blood samples, serum aminotransferases and glutamate dehydrogenase were determined using optimized UV methods [24, 25].

### 2.5. Histological Study

At the end of the experimental design, the animals were decapitated without previous sedation. Then they were bled and their liver and kidneys were removed and prepared for structural and ultrastructural studies. 

Small portions of the kidney and liver previously separated were washed with physiological solution, fixed in a 10% formaldehyde solution, and embedded in paraffin. They were cut into 4-5 *μ*m thick sections and stained with hematoxylin-eosin [26]. The histological slices were observed under an Axiostar plus Zeiss optical microscope. 

For observation under the electron microscope, the liver and kidney portions were processed as follows: they were fixed in a 3.5% glutaraldehyde solution in 0.1 M phosphate buffer at pH 7.40 for 3 h and then placed in 1% osmium tetroxide. Then, the samples were treated with an aqueous solution of 2% uranyl acetate for 40 min. After fixation, the tissues were gradually dehydrated by a successive passage through a series of increasing alcohol concentrations. Finally, they were passed through ketone and embedded in resin. Ultrathin sections were treated with uranyl acetate, placed in citrate, and examined at 50 kV with a Zeiss EM 109 transmission electron microscope belonging to LAMENOA - Electron Microscopy Laboratory of Northwestern Argentina [27].

### 2.6. Statistical Analysis

Analysis of variance and later comparison of means were performed with Tukey's test (alpha = 5%).

## 3. Results

During the 120-day chronic assay, the animals in the different groups increased their weight and maintained their healthy status. None of them died during the treatment.

Hepatosomatic ratio was determined at the end of the treatment, after the animals had been sacrificed. No significant differences were found in groups B, C, or F with respect to control group A. Groups D and E showed a statistically significant decrease in the hepatosomatic ratio with respect to control group A. These results are shown in Table 1. 

**Figure 1:**
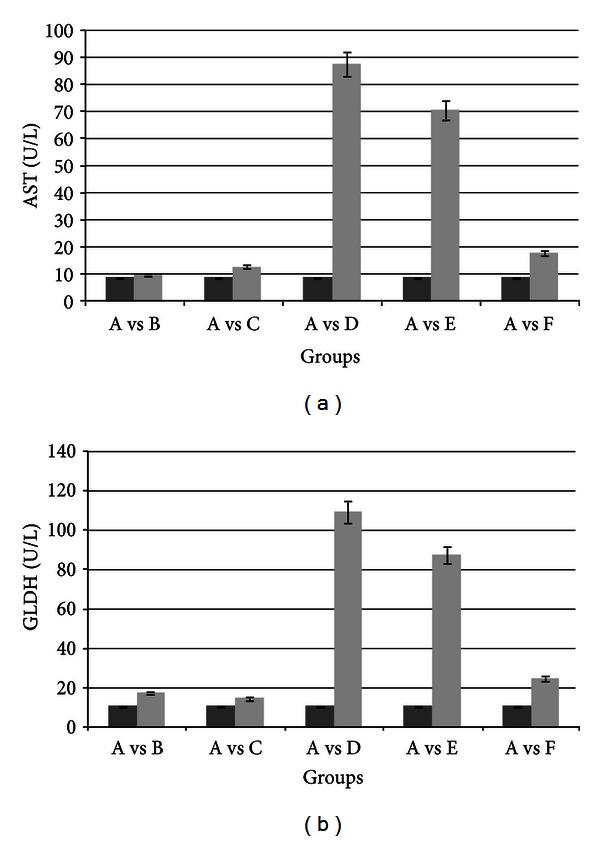
Serum AST and GLDH in treated animals compared with controls. Treatment with CyA 25 mg/kg/day increased AST and GLDH activity (AvsB). The animals treated with Vit E 10 mg/Kg/day showed significant increases in GLDH and AST activity (AvsE). The animals pretreated with TMZ and TMZ + CyA showed no significant changes in this activity (AvsF). SD is shown at the top of each column.

Figure 1 shows the statistically significant increase in the activity of mitochondrial enzymes AST and GLDH. This increase was observed after chronic treatment with 25 mg/Kg/day CyA. 

These biochemical findings show the experimental nephrotoxicity and hepatotoxicity induced by CyA. Group F, pretreated for 20 days with TMZ and then for 120 days with TMZ + CyA, showed a protective effect on the liver profile, with enzymatic activity of AST and GLDH comparable to that in control group A. However, the therapeutic scheme applied to group E reflected a behavior similar to group D. Vitamin E, in these conditions, did no exert a hepatoprotective effect. In order to relate the biochemical changes observed to the histoarchitecture of the liver parenchyma, we studied histological slices with Mallory and hematoxylin-eosin staining of the liver parenchyma.

**Figure 2:**
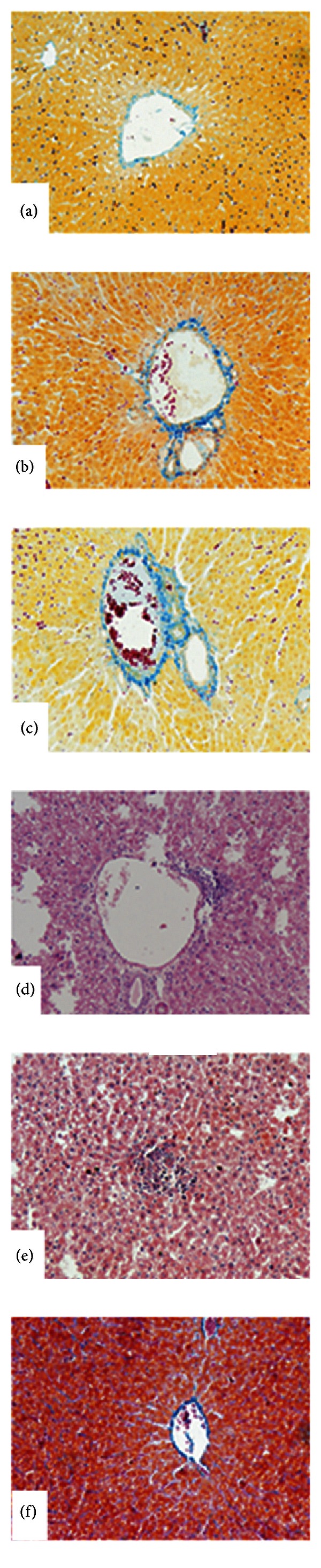
Histoarchitecture of the liver parenchyma. Hematoxylin-eosin (20x). (a) Control group A (10x). (b) Control group of treatment with vitamin E (40x). (c) Control group of treatment with Trimetazidine (40x). (d) Control group of treatment with 25 mg/Kg/day of CyA (20x). (e) Group with synergistic treatment 10 mg VitE/Kg/day + 25 mg CyA/Kg/day (20x). (f) Group pretreated with 20 mg of TMZ and synergistic treatment with 20 mg TMZ/Kg/day + 25 mg CyA/Kg/day for 120 days (40x).

In Figure 2(a), 10x magnification shows hepatocytes in cords disposition around the central vein. They present a homogeneous cytoplasm with a central nucleus, with no cytological alterations. 

In Figures 2(b) and 2(c), (20x) control groups: B with Vitamin E and C with TMZ, respectively, shows liver parenchyma histoarchitecture with no alterations. In Figure 2(d) (20x) shows the histological slice of the liver parenchyma of group D, treated with 25 mg/Kg/day CyA for 120 days. We can see the central vein and the infiltration of mononuclear elements in the perivascular space. In Figure 2(e) (20x) shows the histological slice of the liver parenchyma of group E, treated with 25 mg CyA/Kg/day and 10 mg Vit E/Kg/day for 120 days. We can see mononuclear infiltrates in the whole liver parenchyma, including the portal spaces.

In Figure 2(f) (40x) shows the protective effect on liver parenchyma of pretreatment with TMZ for 20 days and then for 120 days with 20 mg TMZ/Kg/day + 25 mg CyA/Kg/day. We can see the conserved liver parenchyma histoarchitecture, similar to the one in animals control group A.

Portions of the liver were processed for ultrastructural observation, especially of the mitochondria. 

**Figure 3:**
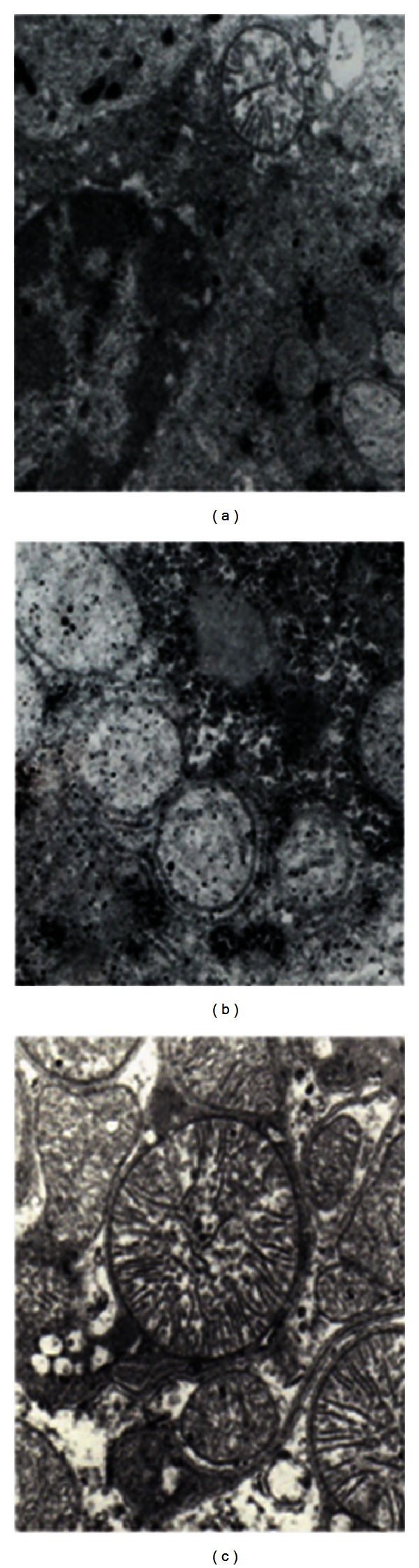
Ultrastructure of hepatocytes of the different groups. (a) Control group A (7800x). (b) Control group of treatment with 25 mg/Kg/day of CyA (82.640x). (c) Group pretreated with 20 mg of TMZ and synergistic treatment with 20 mg TMZ/Kg/day + 25 mg CyA/Kg/day for 120 days (82.640x).

Figure 3 shows the electron microphotograph of hepatocytes of the different treated groups with 82.640x magnification.

Figure 3(a) corresponds to control group A. We can see a hepatocyte with its nucleus with lax chromatin. At the top a mitochondria with normal size and structure can be observed in a transverse section. The intramitochondrial crests can be clearly seen (7800x).

Figure 3(b) shows the electron microphotograph of a hepatocyte from group D. The animals treated with 25 mg CyA/Kg/day for 120 days show swollen mitochondria with destruction of the inner membrane and of their intramitochondrial crests, where enzyme complexes I and II are located (82.640x). 

Figure 3(c) shows the electron microphotograph of a hepatocyte from group F. The animals pretreated with 20 mg of TMZ and then with 20 mg TMZ/Kg/day + 25 mg CyA/Kg/day for 120 days show conserved mitochondrial ultrastructure. Intramitochondrial crests can be observed (82.640x).

**Figure 4:**
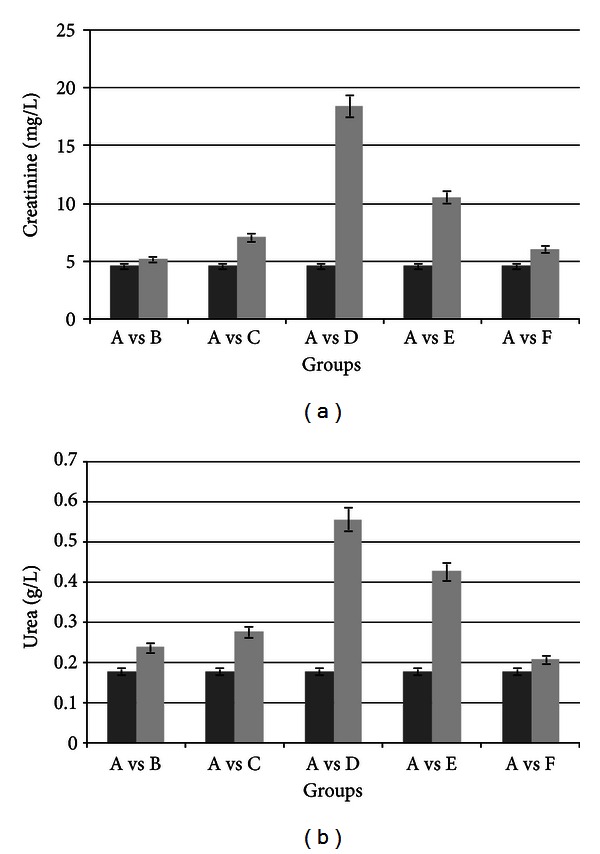
Serum urea in treated animals compared with controls. Animals treated with 25 mg CyA/kg/day show a significant increase in urea and creatinine (AvsD). Group E shows a behavior similar to group D (AvsE). Pretreatment with TMZ and then with TMZ + CyA show the protective effect of TMZ on the renal function (AvsF). SD at the top of the columns.

 The renoprotective effect was studied.  Figure 4 shows a statistically significant increase in the levels of serum urea and creatinine in animals from groups D (AvsD). These biochemical findings show the experimental nephrotoxicity induced by the treatment with CyA at a dose of 25 mg/Kg/day.

The therapeutic scheme used with group F (pretreatment with 20 mg TMZ/Kg/day and simultaneous treatment with 20 mg TMZ/Kg/day + 25 mg CyA/Kg/day) did not show significant biochemical changes (AvsF), showing the protector effect of TMZ on the renal function.

 The nitrogen compounds urea and creatinine in group E were within the ranges of group D. Vit E in these conditions did not exert a renoprotective effect.

In both control and treated groups diuresis was measured for 24 h, no statistically significant differences being found (data not shown). In order to compare the biochemical changes with the histoarchitecture of the renal parenchyma, histological slices of the kidney of rats from the different groups were studied. The following figures show these slices under optical and electron microscopes.

**Figure 5:**
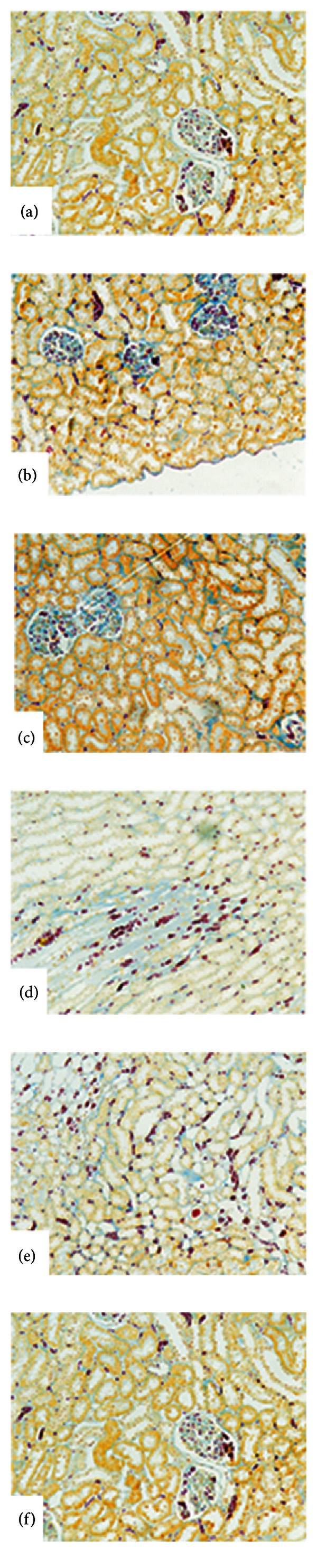
Histoarchitecture of the renal parenchyma. Hematoxylin-eosin (20x). (a) Control group A. (b) Control group of treatment with Vitamin E (20x). (c) Control group of treatment with Trimetazidine (20x). (d) Group with synergistic treatment 10 mg VitE/Kg/day + 25 mg CyA/Kg/day (20x). (e) Group pretreated with 20 mg of TMZ and synergistic treatment with 20 mg TMZ/Kg/day + 25 mg CyA/Kg/day for 120 days (20x).

Figure 5 shows histological slices with Mallory staining of the renal parenchyma.

Figure 5(a) shows the kidney of a rat from control group A (10x). The renal cortex with a central glomerulus can be seen as well as a well-defined Bowman's space. The rest of the parenchyma shows transverse sections of proximal and distal convolute tubules.

Figure 5(b) shows a histological slice of group B (20x). Kidney of rat was treated with 10 mg VitE/Kg/day. Part of a conserved glomerulus can be seen; however, in the tubular epithelium can be seen cell desquamation and hydropic degeneration corresponding to revertible dysplasia. 

Figure 5(c) shows the kidney of a rat treated with 20 mg TMZ/Kg/day (20x). The renal cortex with conserved glomerulus and tubules can be seen.  Figure 5(d) corresponds to the kidney of a rat treated with 25 mg CyA/kg/day (20x). In the renal medulla can be seen an interstitial fibrous.

Figure 5(f) shows the kidney of a rat pretreated with 20 mg TMZ/kg/day and then with 20 mg TMZ/Kg/day + 25 mg CyA/Kg/day belonging to group F (10x). The renal cortex with glomeruli and a transverse slice of the conserved tubules can be seen, showing the protector effect of TMZ on the renal parenchyma.

However, the therapeutic scheme applied to group E reflected a behavior similar to group D. Vit E in these conditions did not exert a renoprotective effect (Figure 5(e)).

Sections of the kidney were processed for ultrastructural observation, especially of the mitochondria. 

**Figure 6:**
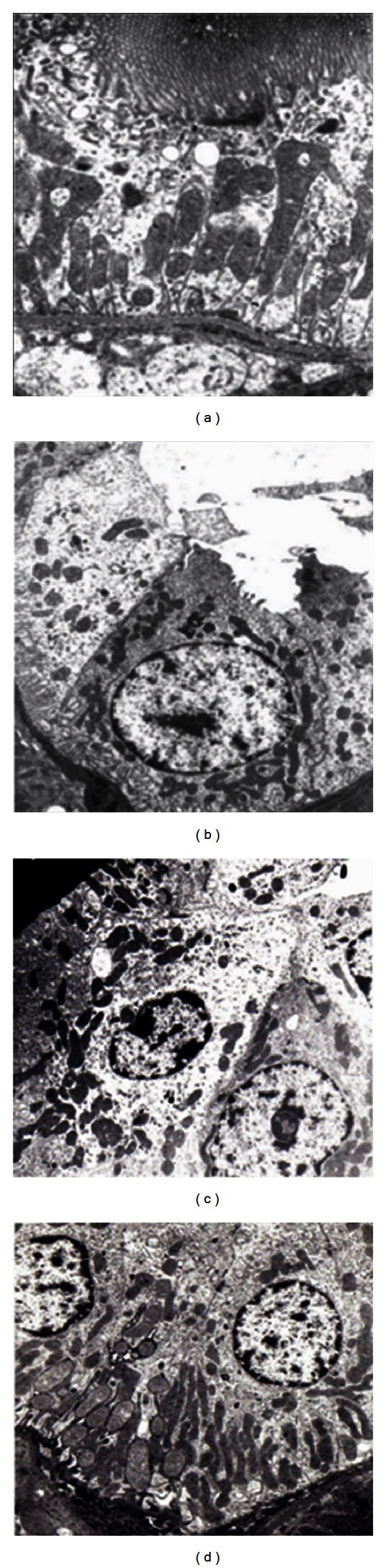
Ultrastructure of the tubule renal cell-proximal convolute tubule (7.800x): (a) Control group A. (b) Group D treated with 25 mg CyA/Kg/day for 120 days. (c) Group E with synergistic treatment 25 mg CyA/Kg/day + 10 mg Vitamin E. (d) Group F pretreated with 20 mg of TMZ and synergistic treatment with 25 mg CyA/Kg/day + 20 mg of TMZ for 120 days.

Figure 6 shows the ultrastructure of transverse slices of proximal convoluted tubules.

Figure 6(a) shows the normal ultrastructure of the renal tubular epithelium of the male Wistar rats; can be seen cells with apical pole showing brush border conserved. In the apical brush border, can be seen the microvilli typical of the proximal tubular epithelium (7.800x).

Figure 6(b) shows the tubular epithelium of group D, treated with 25 mg CyA/Kg/day. In the transverse section of the epithelium can be seen a tubular cell with intact basement membrane and atrophied apical border, and next cell without nucleus. Both cells show disarranged mitochondria, corresponding to cytoplasmic vacuolization or hydropic degeneration (7.800x).

Figure 6(c) shows two tubular cells from group E, treated with 10 mg VitE/Kg/day + 25 mg CyA/Kg/day. Both cells have their respective nucleus and cytoplasms conserved. However, can be seen mitochondria with altered shapes, sizes, and arrangement in relation to the control group. On the upper right hand side we can see the apical border totally atrophied and with no microvilli (7.800x).

Figure 6(d) shows two nucleated tubular cells from group F, pretreated with 20 mg TMZ/Kg/day and then with 20 mg TMZ/Kg/day + 25 mg CyA/Kg/day. Can be seen the intact basement membrane with the perpendicular arrangement of the mitochondria at the basal domain. 

## 4. Discussion

CyA is the drug most frequently used in transplant surgery because of its potent immunosuppressive action. However, its clinical use is accompanied by adverse side effects such as hypertension, nephrotoxicity, and hepatotoxicity. Previous studies established that ROS production and oxidative stress situation are involved in CyA cytotoxicity in cultured rat hepatocytes. In all cases, they attribute to CyA the ability to produce oxidative stress. However, none of them have explained what is the mechanism of formation and accumulation of ROS [11–15]. However, It has been demonstrated in numerous *in vivo *and *in vitro *experiments that CyA-induced renal failure and increased the synthesis of ROS, thromboxane, and lipid peroxidation products in the kidney [28, 29].

Different authors published works about the benefits of treatment with vitamin E to attenuate the nephrotoxic and hepatotoxic effects of CyA. Most of them have based their conclusions on the antioxidant capacity of alpha tocopherol. In a work published in 2007 [28, 29], oxidative stress and mitochondrial dysfunction were studied in tubular renal cells *in vitro*. The authors concluded that the cellular toxicity of CyA resulted from important alterations in the mitochondrial physiology and structure, with an increase in ROS synthesis and a decrease in antioxidant capacity. 

Other authors have reported vitamin E as a protector against CyA-induced cytotoxicity in a rat hepatocyte culture [30, 31]. One of those reports [30], with *in vitro* experiments, concluded that there is an imbalance in antioxidant enzymes due to the direct action of CyA on the primary hepatocyte culture and the fact that the simultaneous treatment with CyA and Vit E reduces oxidative stress, inhibiting lipoperoxidation and restoring antioxidant enzymes.

In previous papers, we reproduced in animals the therapeutical schemes used with human transplant recipients. We have experimentally demonstrated that nephrotoxicity and hepatotoxicity are the main secondary effects of CyA treatment. These effects are time period and dose dependent [7–9].

For the purpose of finding a drug that would exert a protective effect against CyA-induced hepato- and nephrotoxicity, we assayed two drugs: vitamin E and TMZ.

We conclude that vitamin E does not exert a protector effect against CyA-induced nephrotoxicity. We base these results on the increased levels of serum urea and creatinine in group E, shown in Figure 4. These results correlate with our structural and ultrastructural findings (Figures 5(d) and 6(c)). 

Special attention should be paid to the effect of treatment with 10 mg/Kg/day Vit E on group E. In Figure 5(b), we can see part of a conserved glomerulus and in the tubular epithelium cell desquamation and hydropic degeneration can be observed. These morphological changes correspond to regenerative dysplasia. 

 Apparently, Vit E by itself is capable of producing morphological changes in the tubular renal cell. Some authors have reported that when the optimal level (80 *μ*g/dL) of this antioxidant is surpassed, the risks of toxicity increase [12].

 On the other hand, the hepatotoxic effect of CyA is not attenuated by treatment with vitamin E. If we analyze Table 1, we can see that group E shows a statistically significant decrease in hepatosomatic ratio comparable only to group D with treatment with 25 mg CyA/Kg/day. This decrease correlates with the increase in the activities of mitochondrial sublocalization enzymes: AST and GLDH, in Figure 1. The increase in AST and GLDH is statistically significant with respect to the other control and treated groups.

These results are corroborated by optical microscopy of the liver parenchyma in Figure 2(e). We can see infiltrates of mononuclear elements in the whole liver parenchyma, including the portal spaces, a similar histoarchitecture to the one described in group D treated with 25 mg CyA/Kg/day. Figure 2(d).

To find a protective drug against those effects we assayed the cardioprotector Trimetazidine (TMZ). Some authors have reported that TMZ exerts a cytoprotective effect on the cardiomyocyte [16–20]. Our previous works have demonstrated the cytoprotection of TMZ in an *in vivo* model of gentamicin-induced nephrotoxicity [21].

 In the present work we studied and described the cytoprotective effect of TMZ against CyA-induced toxicity on the liver and kidney of treated rats. We demonstrated that pretreatment with 20 mg/Kg/day TMZ followed by treatment with 20 mg/Kg/day TMZ + 25 mg/Kg/day CyA for 120 days preserves the morphofunctionality of the tubular renal cell and of the liver cell. This was clearly manifested by the improvement in all the biochemical variables determining CyA-induced hepato- and nephrotoxicity (Figures 1 and 4).

These biochemical parameters correlate with the micro- and ultrastructural characteristics of the liver parenchyma. We can see histoarchitecture similar to the one in control group A (Figure 2(f)). Hepatocyte mitochondria ultrastructure of animals in group F appears conserved. We can see the intramitochondrial cristae, site of the mitochondrial respiratory chain (82.640x) Figure 3(c).

On the other hand, we demonstrated in an *in vivo* animal model to the mitochondria as a target for the toxic action of CyA. We reported that chronic treatment with CyA at doses above 15 mg/Kg/day causes alterations in the mitochondria, by the functional alteration in the mitochondrial respiratory chain in its complexes I and II, since this dose- and time-dependent effect requires active cell metabolism [9].

In our study we investigated whether TMZ allows inhibition of the mitochondrial alteration induced by CyA. Trimetazidine protects the cells against the changes produced by the CyA-induced oxygen deficit. TMZ could offset ATP synthesis caused by the chronic administration of CyA. Trimetazidine optimizes the energetic metabolism of the ischemic cell through a metabolic exchange (“switch”) between the fatty acid and glucose oxidation. In theory, TMZ would reduce strongly the fatty acid oxidation towards glucose, without affecting the mitochondrial respiratory chain efficiency. Furthermore, TMZ increases the production of phospholipids in the mitochondrial membranes, which confers stability to these structures. 

We conclude the following.

- The target of the cytotoxic action of CyA is in enzymes of complexes I and II of the mitochondrial respiratory chain, which results in an alteration in the energy metabolism and leads to cell death by necrosis.
- TMZ exerts a cytoprotective effect both on the liver cells and on the tubular renal cells. Among the different mechanisms of action proposed by some authors, pretreatment with TMZ would contribute to optimizing the energy metabolism of the mitochondria treated with TMZ + CyA at the doses and time periods studied.
- Pretreatment with TMZ is essential for the protection of the cell since it confers stability and prevents the incorporation of CyA into the inner membrane of the mitochondria.
- The antioxidant vitamin E does not exert a protective effect since we demonstrated that the CyA action in the mitochondrial chain does not involve ROS.

## Figures and Tables

**Table 1:** Hepatosomatic ratio in the treated groups.

Group	Hepatosomic ratio
A (control)	3.62 ± 0.06
B (control Vit E)	3.22 ± 0.08
C (control TMZ)	3.50 ± 0.08
D (CyA)	2.25 ± 0.05
E (Vit E + CyA)	2.30 ± 0.06
F (TMZ + CyA)	3.28 ± 0.08

Hepatosomic ratio: (liver weight × 100)/total body weight.
